# Quantitative evaluation of the medicine innovation policy in China: based on the PMC-Index model

**DOI:** 10.3389/fpubh.2024.1403320

**Published:** 2024-05-16

**Authors:** Dan Guo, Liwen Qi, Xiaoting Song

**Affiliations:** Shanghai International College of Intellectual Property, Tongji University, Shanghai, China

**Keywords:** PMC-Index model, medicine innovation policy, China, quantitative evaluation, policy formulation

## Abstract

**Introduction:**

Medicine innovation is crucial in promoting the sustainable development of medicine undertakings, which has significant economic and social benefits. China is the main force in global medicine consumption, with a huge demand for innovative medicines. Thus, the Chinese government releases a series of policies aimed at providing scientific and reasonable guidance for medicine innovation. However, there is inadequate quantitative evaluation and comparison of various medicine innovation policies in the existing studies.

**Methods:**

This paper adopts the approach of text mining and the Policy Modeling Consistency Index (PMC-Index) model to construct an evaluation system and then quantitatively evaluates and compares the traditional Chinese medicine innovation policies (TCMIPs), the biological medicine innovation policies (BMIPs), and the multiple medicine innovation policies (MMIPs) in China.

**Results:**

The results indicate that: (1) The three types of drug innovation policies have similarities in content and goal through comparative analysis of high-frequency words, while they also have their own characteristics. (2) The average PMC-Index of 29 TCMIPs is 5.77, which has the highest policy bad rate (21%); the average PMC-Index of 12 BMIPs is 6.21, which has the highest policy good rate (92%); moreover, the average PMC-Index of 35 MMIPs is 6.06, which has the highest policy excellence rate (26%). (3) The BMIPs, MMIPs, and TCMIPs have similar scores on policy object, policy orientation, policy timeliness, policy evaluation, and policy accessibility, while they differ significantly mainly on policy nature, incentive method, policy function, policy issuing agency, and policy instrument.

**Discussion:**

This study contributes to a comprehensive understanding of medicine innovation policies in China, in order to provide theoretical support for future policy formulation and optimization in the medicine industry. Moreover, we expand the application scenarios of policy diffusion theory.

## 1 Introduction

Medicine plays an important role in safeguarding human health and promoting medical progress (1). Innovative medicine is the frontier force of the pharmaceutical industry, which has huge social and economic benefits (2). Cancer and autoimmune diseases have become major health challenges globally, while innovative medicines can improve the cure rate of these difficult diseases (3). In 2022, the global market size of innovative medicines reached 1,027 billion dollars, accounting for approximately 69.5% of the global pharmaceutical market. However, the scale of the innovative medicine market in China is only 142 billion dollars in 2022. Moreover, due to a lack of competition among similar high-quality medicines, the prices of imported innovative medicines have remained high for a long time in China. Before the domestic programmed death-1 was available, patients spent about 70,000 to 80,000 dollars annually on imported innovative medicines such as Keytruda and Opdivo (4). However, the treatment cost for patients decreases to less than 7,000 dollars when domestic innovative medicines launch. Domestic innovative medicines are gradually replacing imported medicines, which contributes to controlling health insurance expenditures and reducing the burden on patients. Furthermore, China is aging much faster than the global average. The proportion of older adult people aged 65 years and older in China doubled to 14.2% from 2000 to 2021 (5). More and more older adult people mean a growing market space for the innovative medicine industry, and the demand for innovative medicines will continue to increase in the future. How to stimulate medicine innovation in China is significant and urgent, so the Chinese government has formulated a large number of medicine innovation policies.

These medicine innovation policies include development plans, guidelines, and implementation opinions to support and encourage the development of the medicine industry. This indicates that the government is highly concerned about public health (6, 7). Reviewing the medicine innovation policies in China, what are the similarities and differences among these policy texts? What are the overall quality and individual characteristics of the medicine innovation policy in China? How can we identify the strengths and weaknesses of medicine innovation policy design and provide targeted improvement strategies? Academics have yet to answer these questions. Thus, the motivation of this study is to assess medicine innovation policy in China, answer the above research questions, and help policymakers improve the medicine innovation system to promote medicine innovation development. However, the medicine innovation policy in China lacks a comprehensive and scientific method for evaluating the advantages and disadvantages of policies. There are various methods for policy evaluation (8–10), but the more cutting-edge at present is the PMC-Index model (11). The PMC-Index is a policy evaluation methodology proposed by Estrada in 2011 that assesses the internal consistency of policies in several dimensions and identifies the advantages and disadvantages of each policy (12). This paper attempts to use the PMC-Index method to quantitatively assess the consistency level of medicine innovation policies in China.

**Figure 1:**
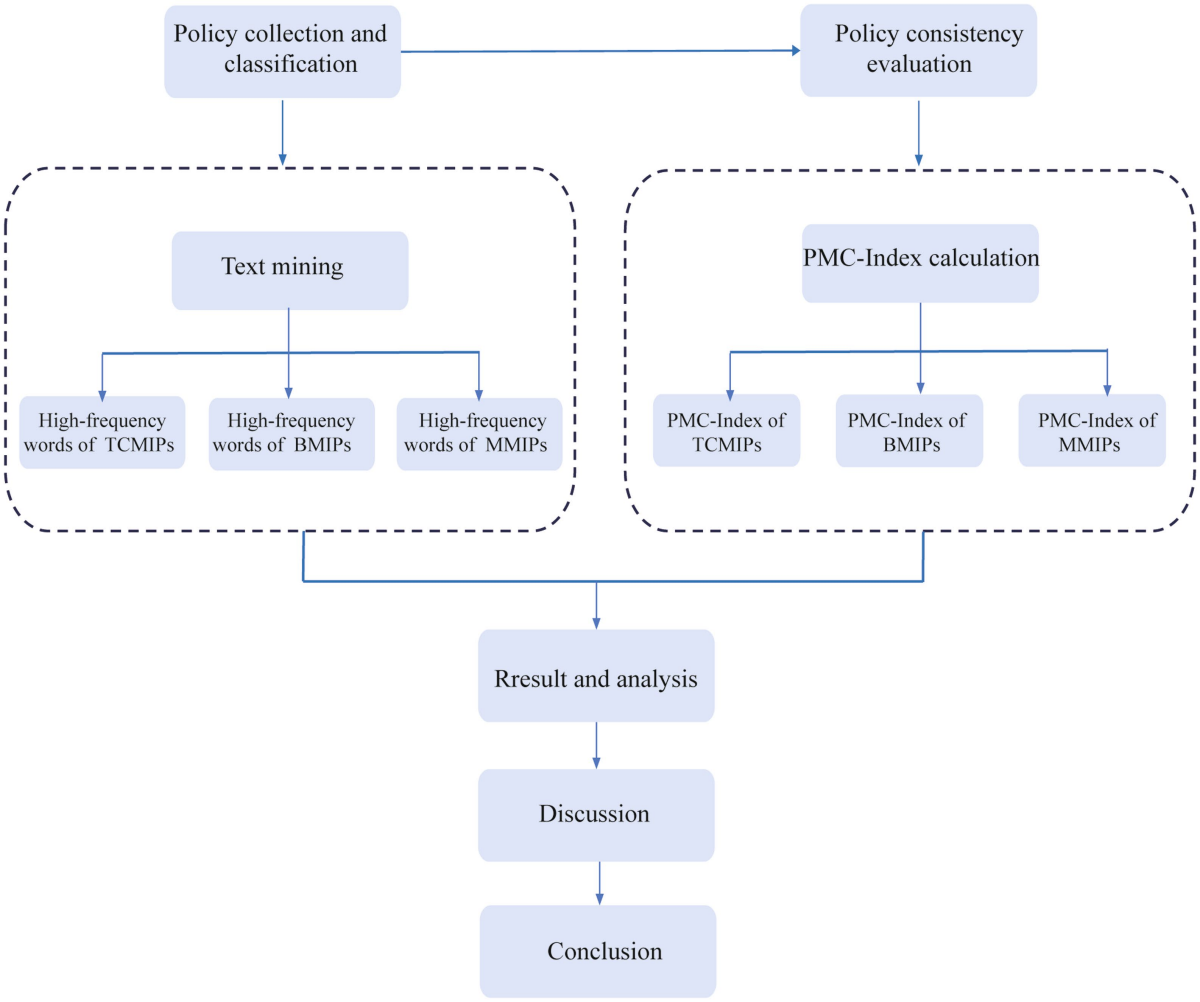
Research framework.

This study attempts to fill the gaps in the existing literature, and our major contributions are as follows: (1) There is insufficient comparative analysis of medicine policies in the existing literature, with some studies focusing on a single industry (13). However, this study conducts comparative analysis and selects comprehensive samples, including the traditional Chinese medicine innovation policies (TCMIPs), the biological medicine innovation policies (BMIPs), and the multiple medicine innovation policies (MMIPs) issued by the Chinese government; MMIPs cover the innovation of traditional Chinese medicine, biological medicine, and chemical medicine at the same time. Since the retrieved policies relate to the innovation of chemical medicines are technical guidelines without specific planning content, our study excludes policies that relate to chemical medicine innovation in the analysis. (2) Most of the existing literature is about the macro-evaluation of the implementation effect of medicine innovation policies, as it is the endpoint of policy evaluation (14–16), but neglects the analysis of policy content (17–19). However, text mining technology is adopted in this study to dig deeply into the policy texts of TCMIPs, BMIPs, and MMIPs so as to identify the basic elements and the internal logic of various medicine innovation policies. Moreover, this paper constructs the PMC-Index model to quantitatively evaluate and compare the TCMIPs, BMIPs, and MMIPs, respectively, which provides theoretical support for future policy formulation in the medicine industry. The research framework is illustrated in Figure 1.

The rest of this study is organized as follows: Section 2 reviews the literature on medicine innovation and policy evaluation, and identifies the shortcomings in existing research. Section 3 describes the research design, including sample selection and methodology. Section 4 reports the quantitative analysis results of TCMIPs, BMIPs, and MMIPs and proceeds with a comparative analysis and discussion of various policies. Section 5 summarizes this study and elaborates on the limitations.

## 2 Literature review

To promote better practice of medicine innovation policies, scholars have studied these policies from both quantitative and qualitative perspectives. The quantitative research regarding medicine innovation policies mainly focuses on their implementation effects evaluation, while the qualitative research regarding these policies chiefly concentrates on their policy content evaluation. Compared with the qualitative research, the quantitative research on medicine innovation policies is more abundant. That is, most existing literature focuses on the implementation effectiveness of medicine innovation policies. For example, Bouet (20) utilized probit and logit techniques to evaluate the effect of TRIPs on Indian pharmaceutical industry innovation. Gamba (21) employed the zero-inflated negative binomial model to analyze the medicine intellectual property protection reform policies in developed and developing countries and believed that medicine innovation is highly sensitive to intellectual property protection. Aghmiuni et al. (22) discussed the supportive innovation policies in biological medicine and found that these policies have a significant impact on the development of biological medicine innovation. Moreover, existing studies also indicate that some medicine innovation policies in China contribute to innovation quality. For example, Liu et al. (23) applied the difference-in-differences model to find that the generic consistency evaluation policy has a positive impact on the innovation quality of the Chinese pharmaceutical industry. Gu et al. (24) adopted listed medicine companies in the Shanghai and Shenzhen A-share markets as samples and empirically found that centralized drug procurement policy has a significant improvement on the quality of medicine innovation. In addition, the qualitative evaluation of medicine innovation policies in existing research mainly concentrates on the specific interpretation of policy content. Doran et al. (25) reviewed the reforms of medicine innovation policies in Australia and elaborated on the impact of price control on innovation. Karampli et al. (26) outlined the study findings on the impact of medicine innovation on medicine expenditure growth and described the challenges faced by Greek drug innovation policies. Liu et al. (27) described the medicine innovation policies to accelerate medicine review and approval in China, stating that the development of innovative medicine benefits from these accelerated policies. Overall, the above literature helps researchers understand medicine innovation policies from different perspectives and provides insights for policy optimization. However, these studies have mainly examined medicine innovation policies at the macro level, lacking a systematic evaluation of medicine innovation policies in China. Thus, research on medicine innovation policies in China has yet to be expanded.

Policy evaluation is a complex and systematic program that plays a crucial role in guiding policy formulation and optimization (28–30). Choosing appropriate and scientific evaluation methods is the foundation of policy evaluation. The existing studies have proposed a variety of methods to evaluate policies, such as the five kinds of evaluation tools (31), the “3e “assessment framework (32), the index of legal changes (33), hierarchical analysis (34), Delphi method (35), content analysis method (36), and difference in difference analysis (37–39). As previously stated, the five kinds of evaluation tools, the “3e “assessment framework, and the index of legal changes are relatively outdated and one-sided in their assessment of policy. Moreover, hierarchical analysis, the Delphi method, and the content analysis method have more subjective evaluation processes (40). Furthermore, the difference in difference analysis focuses on evaluating the implementation effect of a certain policy (41–43), lacking systematic evaluation of a series of policies (44). These above policy evaluation methods are widely adopted, but they fall short in terms of objectivity and accuracy. In addition, these policy evaluation methods pay less attention to individual differences and the texts of policies. However, the PMC-Index model combines qualitative and quantitative approaches in a more comprehensive and objective way than the above methods, which are widely used to evaluate policies. It can provide an overall evaluation of policy consistency as well as systematically analyze individual policy differences from various dimensions. In previous literature, research on the PMC-Index model has been on the rise, and many satisfactory results have been achieved. For example, Liu et al. (45) utilized the PMC-Index model to discuss the power battery recycling policies of the central and local governments and found that the policymaking ability of the central government is stronger. Fan et al. (46) employed the PMC-Index model to investigate China’s municipal solid waste policies and identified that these policies are generally reasonable. Zhao et al. (47) utilized the PMC-Index model to explore energy security in China and believed that the administrative level of the issuing agency positively affects the PMC-Index. In addition, many studies have used this model for policy evaluation, including fire safety education policy (48), traditional Chinese medicine development policy (13), new energy vehicle policy (49, 50), and internet healthcare policy (51). These studies reflect that the PMC-Index model has good applicability for opening up the black box of policy formulation and promoting policy evaluation.

Overall, there is extensive literature on medicine innovation and policy evaluation, while the existing research still has some shortcomings. First, there is much literature on the implementation performance of medicine innovation policies, while little literature evaluates these policies from a policy formulation perspective. Second, the existing literature has not yet applied the PMC-Index model to evaluate medicine innovation policies, lacking the inclusion and comparative study of TCMIPs, BMIPs, and MMIPs simultaneously. Hence, this paper aims to narrow these gaps by investigating the TCMIPs, BMIPs, and MMIPs, constructing an evaluation indicator system, and utilizing the PMC-Index model to analyze these policies. The goal of this study is to gain insights into the current status of various medicine innovation policies and provide references for the formulation and improvement of these policies in the future.

## 3 Research design

### 3.1 Data sources and samples selection

The medicine innovation policies issued by the Chinese government are taken as the research object in this study. To obtain the policy texts on the medicine innovation policies systematically, we adopt three search paths. Firstly, relevant policy documents are retrieved on the portals of the State Council (SC), the National Health Commission (NHC), the National Medical Products Administration (NMPA), the National Administration of Traditional Chinese Medicine (NATCM), and other related government departments. Secondly, we search for relevant policy documents on the Peking University Law Website.^1^ Finally, search platforms such as Baidu and Google are used as supplements for policy document collection. We set search terms such as “medicine innovation,” “TCM innovation,” “biological medicine innovation,” and “chemical medicine innovation” in these databases. Considering the evolution characteristics of China’s medicine innovation policies, the retrieval period is from 2000 to 2023. Due to some repeated and invalid collection, the policy documents are screened according to the following principles: (1) only the national-level policy documents are selected in this study; (2) we eliminate some documents that have been revised or repealed; (3) policy documents such as working arrangements, letters, technical guidelines, and approvals are excluded; and (4) we focus on policies with specific plans. After eliminating the irrelevant policies, 76 policy documents are obtained, including 29 TCMIPs, 12 BMIPs, and 35 MMIPs (some policies are shown in Table 1). These policy documents mainly cover laws, regulations, plans, outlines, notices, and other relevant rules on medicine innovation in China.

**Table 1:** The 29 TCMIPs, 12 BMIPs, and 35 MMIPs (partial).

Level	Code	Policy name	Issuing agency	Date issued
TCMIPs	P1	The Tenth Five-Year Plan for TCM	NDRC,	2001.09.04
	P2	Outline of TCM modernization development	NHC; NMPA; NATCM, etc.	2002.10.10
	……	……	……	……
	P28	Notice on issuing the Implementation Plan of the Major Project for the Revitalization and Development of traditional Chinese Medicine	SC	2023.02.10
	P29	Announcement on the promulgation of the Special Provisions on the Administration of Registration of Chinese Medicine	NMPA	2023.02.10
BMIPs	Q1	Announcement on the implementation of high-tech projects in the biotechnology industry during the Tenth Five-Year Plan Period	NDRC	2002.02.11
	Q2	Announcement on the organization and implementation of the special project of biomedical engineering high-tech industrialization	NDRC	2003.02.11
	……	……	……	……
	Q11	The 14th Five-Year Plan for bioeconomic development	NDRC	2021.12.20
	Q12	Biosecurity Law of the People’s Republic of China	NPC Standing Committee	2020.10.17
MMIPs	R1	China’s pharmaceutical industry “tenth Five-Year plan”	Department of Industry Planning, State Economic and Trade Commission	2001.10.10
	R2	Medical science and technology policy	MST; NATCM	2002.09.18
	……	……	……	……
	R34	Implementation Opinions on Comprehensively Strengthening drug regulatory capacity building	SC	2021.04.27
	R35	Notice on the issuance of the 14th Five-Year Plan for the modernization of Market Supervision	SC	2021.12.14

### 3.2 Identification of the policy text features

Before the construction of the PMC-Index model, ROSTCM 6 software is adopted for text mining of the above policies (45, 48). We process the policy documents using the ROSTCM6 software, including policy integration, word segmentation, and high-frequency word statistics. Words that appear more frequently but are meaningless, such as “construct,” “increase,” and “development,” are deleted. Finally, the most relevant and frequent words are extracted for further analysis. High-frequency words can reflect the topic of general interest in policy documents (52). Besides, the top 30 high-frequency words are selected from TCMIPs, BMIPs, and MMIPs, and the Gephi software is adopted to establish a co-occurrence network to clearly show the difference and relevance of various types of medicine innovation policies.

### 3.3 Construction of the PMC-Index model

**Figure 2:**
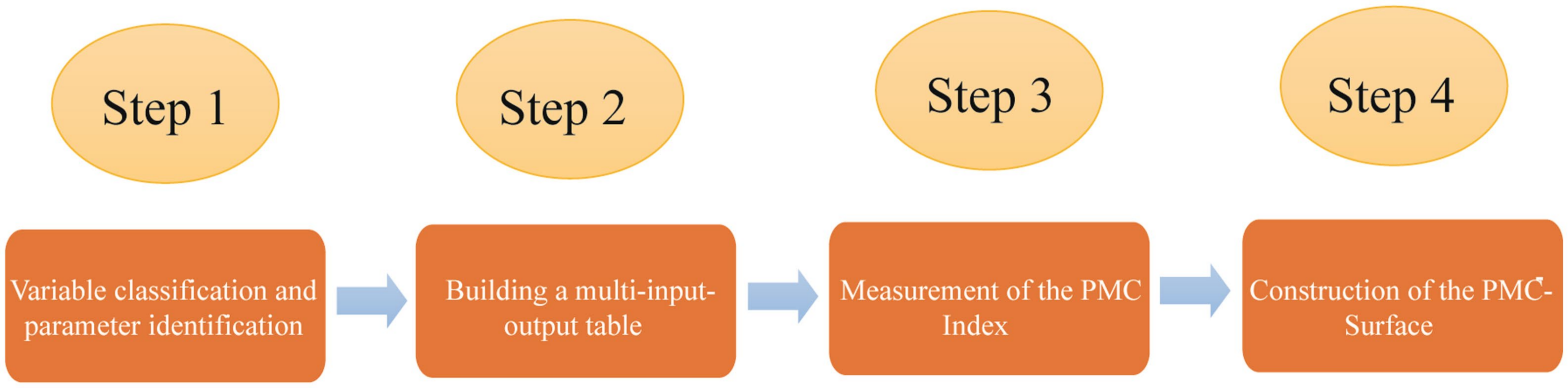
Steps to construct the PMC-Index model.

The PMC-Index model is a scientific and quantitative measurement method for policy evaluation. This model is proposed by Estrada (12), which originates from the Omnia Mobilis hypothesis. The hypothesis believes that everything is in motion and interconnected, so any seemingly irrelevant variable should not be ignored, and the quantity and weights of variables are not restricted. The PMC-Index model analyzes the advantages and disadvantages of each policy and the consistency level of a policy in multiple dimensions by selecting variables comprehensively (47, 53). The PMC-Index model is composed of four main steps (52, 54) (see Figure 2).

#### 3.3.1 Variable classification and parameter identification

The classification of variables and identification of parameters are essential bases for comprehensive policy evaluation. According to the existing studies (45–47) and the specific characteristics of medicine innovation policy, we establish 10 primary variables, namely policy nature (X_1_), policy issuing agency (X_2_), policy object (X_3_), policy timeliness (X_4_), policy instrument (X_5_), policy orientation (X_6_), incentive method (X_7_), policy function (X_8_), policy evaluation (X_9_), and policy accessibility (X_10_). The sub-variables are set for the primary variable by the relevant literature and policy text mining, as shown in Table 2. After classifying, it is essential to identify the parameters of the variables. The binary method is adopted to assign equal weight to all sub-variables (48, 55). If the policy content conforms to the sub-variable, the parameter is set to 1; otherwise, the parameter is set to 0 (46).

**Table 2:** Evaluation index system of the medicine innovation policy.

Primary variables	Sub-variables	Evaluation criteria	References
Policy nature (X_1_)	Prediction (X_11_)	Determine whether the policy reflects prediction.	(12, 55)
	Guidance (X_12_)	Determine whether the policy reflects guidance.	
	Description (X_13_)	Determine whether the policy reflects description.	
	Supervision (X_14_)	Determine whether the policy reflects supervision.	
	Plan (X_15_)	Determine whether the policy reflects plan.	
	Support (X_16_)	Determine whether the policy reflects support.	
Policy issuing agency (X_2_)	SC (X_21_)	Determine whether the issuing agency contains the SC.	(46, 56)
	NHC (X_22_)	Determine whether the issuing agency contains the NHC.	
	MST(X_23_)	Determine whether the issuing agency contains the MST.	
	NMPA (X_24_)	Determine whether the issuing agency contains the NMPA.	
	NATCM (X_25_)	Determine whether the issuing agency contains the NATCM.	
	Other government departments(X_26_)	Determine whether the issuing agency contains other government departments.	
Policy object (X_3_)	Healthcare institution (X_31_)	Determine whether the policy object involves healthcare institution.	(48, 57)
	Administration (X_32_)	Determine whether the policy object involves administration.	
	Research institution(X_33_)	Determine whether the policy object involves research institution.	
	Enterprise (X_34_)	Determine whether the policy object involves enterprise.	
Policy timeliness (X_4_)	Long term (X_41_)	Determine whether the policy content involves more than 5 years.	(47, 58)
	Medium term (X_42_)	Determine whether the policy content involves medium term (3–5 years).	
	Short term (X_43_)	Determine whether the policy content involves (1–3 years).	
	Within 1 year (X_44_)	Determine whether the policy content involves within 1 year.	
Policy instrument (X_5_)	Supply side (X_51_)	Determine whether the SSI is applied in the policy.	(49, 59)
	Environmental side (X_52_)	Determine whether the ESI is applied in the policy.	
	Demand side (X_53_)	Determine whether the DSI is applied in the policy.	
Policy orientation (X_6_)	Encouragement, support (X_61_)	Determine whether the policy has encouragement and support on orientation.	(52)
	Normative guidance (X_62_)	Determine whether the policy has normative and guidance on orientation.	
	Compulsory requirement (X_63_)	Determine whether the policy has Compulsory and requirement on orientation.	
Incentive method (X_7_)	Tax benefits (X_71_)	Determine whether the policy incentive measures involves tax benefits.	(11)
	Investment subsidy (X_72_)	Determine whether the policy incentive measures involves investment subsidy.	
	Intellectual property protection (X_73_)	Determine whether the policy incentive measures involves intellectual property protection.	
	Regulatory and evaluation (X_74_)	Determine whether the policy incentive measures involves regulatory and evaluation	
	Administrative approval incentives (X_75_)	Determine whether the policy incentive measures involves administrative approval incentives.	
Policy function (X_8_)	Talent cultivation (X_81_)	Determine whether the policy function contributes to talent cultivation.	(11)
	Encourage innovations (X_82_)	Determine whether the policy function contributes to encouraging innovations.	
	Industry-academia-research collaboration (X_83_)	Determine whether the policy function contributes to industry-academia-research collaboration.	
	Achievement transformation (X_84_)	Determine whether the policy function contributes to achievement transformation.	
	International exchange (X_85_)	Determine whether the policy function contributes to international exchange.	
Policy evaluation (X_9_)	Clear objectives (X_91_)	Determine whether the policy has clear objectives.	(13)
	Detailed planning (X_92_)	Determine whether the policy is detailed planning.	
	Sufficient basis (X_93_)	Determine whether the policy has sufficient basis.	
	Scientific program (X_94_)	Determine whether the policy has scientific program.	
Policy accessibility (X_10_)	/	Determine whether the policy is accessible to the public.	(45)

#### 3.3.2 Building a multi-input-output table

The multi-input-output table is an analysis framework capable of data storage that evaluates an individual variable in multiple dimensions (60, 61). Building a multi-input-output table is a precondition for the PMC-Index calculation of the medicine innovation policy. In this study, the multi-input-output tables consist of 10 primary variables and 40 sub-variables. The primary variables are not specially ordered and are mutually independent of each other. The sub-variables refine the primary variables in different aspects. In this paper, all researchers analyze and determine whether the policy content involves the sub-variables, respectively. The evaluation results of all researchers are almost the same, apart from a few variables. The controversial variables are further analyzed and discussed collectively on the basis of the policy content and evaluation criteria. After parameter identification, the multi-input-output tables for 29 TCMIPs, 12 BMIPs, and 35 MMIPs are established, as shown in Table 3.

**Table 3:** The multi-input-output table for quantitative evaluation of XX.

Primary variables	X_1_	X_2_	X_3_	X_4_	X_5_	X_6_	X_7_	X_8_	X_9_	X_10_
Sub-variables	X_11_	X_21_	X_31_	X_41_	X_51_	X_61_	X_71_	X_81_	X_91_	/
	X_12_	X_22_	X_32_	X_42_	X_52_	X_62_	X_72_	X_82_	X_92_	
	X_13_	X_23_	X_33_	X_43_	X_53_	X_63_	X_73_	X_83_	X_93_	
	X_14_	X_24_	X_34_	X_44_			X_74_	X_84_	X_94_	
	X_15_	X_25_					X_75_	X_85_		
	X_16_	X_26_								
										

#### 3.3.3 Measurement of the PMC-Index

The PMC-Index calculation includes the specific four steps (46, 55). Firstly, the primary variables and sub-variables are integrated into the multi-input-output tables of TCMIPs, BMIPs, and MMIPs, respectively. Secondly, the binary method is adopted to assign the value of each sub-variable according to text analysis and Eqs 1, 2. Thirdly, the values of 10 primary variables are calculated individually based on Eq. 3. Fourthly, we sum up all primary variables to obtain the PMC-Index of policies according to Eq. 4.


(1) X~N[0,1]

(2) X={XR:[0,1]}

(3) $$X_{i}\left(\sum_{j=1}^{n}\frac{X_{\text{ij}}}{T\left(X_{\text{ij}}\right)}\right)$$

(4) $$PMC=\left[\begin{array}{l} X_{1}\left(\sum_{j=1}^{6}\frac{X_{1j}}{6}\right)+X_{2}\left(\sum_{j=1}^{6}\frac{X_{2j}}{6}\right)+\\ X_{3}\left(\sum_{j=1}^{4}\frac{X_{3j}}{4}\right)+X_{4}\left(\sum_{j=1}^{4}\frac{X_{4j}}{4}\right)+\\ +X_{5}\left(\sum_{j=1}^{3}\frac{X_{5j}}{3}\right)\mathrm{++}X_{6}\left(\sum_{j=1}^{3}\frac{X_{6j}}{3}\right)+\\ +X_{7}\left(\sum_{j=1}^{5}\frac{X_{7j}}{5}\right)+X_{8}\left(\sum_{j=1}^{5}\frac{X_{8j}}{5}\right)\\ +X_{9}\left(\sum_{j=1}^{4}\frac{X_{9j}}{4}\right)+X_{10} \end{array}\right]$$

Where X_i_ refers to the ith primary variable, i = 1, 2, 3, …, 10. X_ij_ refers to the ijth sub-variable, j = 1, 2, 3, …, *n*. T (_Xij_) refers to the number of sub-variables of the ith primary variable.

The PMC indexes of 29 TCMIPs, 12 BMIPs, and 35 MMIPs are calculated based on the above steps. The PMC-Index can evaluate the comprehensiveness and degree of policy consistency. Due to the 10 primary variables selected in our evaluation system, the value of the PMC-Index should be [0, 10]. According to existing studies (12, 52, 55), we classify the values of PMC-Index into 4 evaluation levels (see Table 4).

**Table 4:** Evaluation criteria for policy based on the PMC-Index.

PMC-Index	0 ≤ PMC<5	5 ≤ PMC<7	7 ≤ PMC<9	9 ≤ PMC<10
Evaluation level	Bad	Good	Excellent	Perfect

#### 3.3.4 Construction of the PMC-Surface

The PMC-Surface is constructed to visualize the strengths and weaknesses of policies in multiple dimensions. We plot the PMC-Surface by calculating the PMC matrix. To meet the balance and symmetry of the matrix, X_10_ is left out of this research (11, 46, 47). After removing X_10_, a 3 × 3 matrix is generated by the remaining 9 primary variables, as shown in Eq. 5. Then we utilize the above matrix to draw the PMC-Surface. The concave-convex degree and color depth of the PMC-Surface reflect the strengths and weaknesses of each policy visually. MATLAB software is applied to draw the PMC-Surface diagrams.


(5) $$PMC-\text{surface}=\left[\begin{array}{ccc} X_{1} & X_{2} & X_{3}\\ X_{4} & X_{5} & X_{6}\\ X_{7} & X_{8} & X_{9} \end{array}\right]$$

## 4 Results and analysis

### 4.1 Analysis of high-frequency words

**Figure 3:**
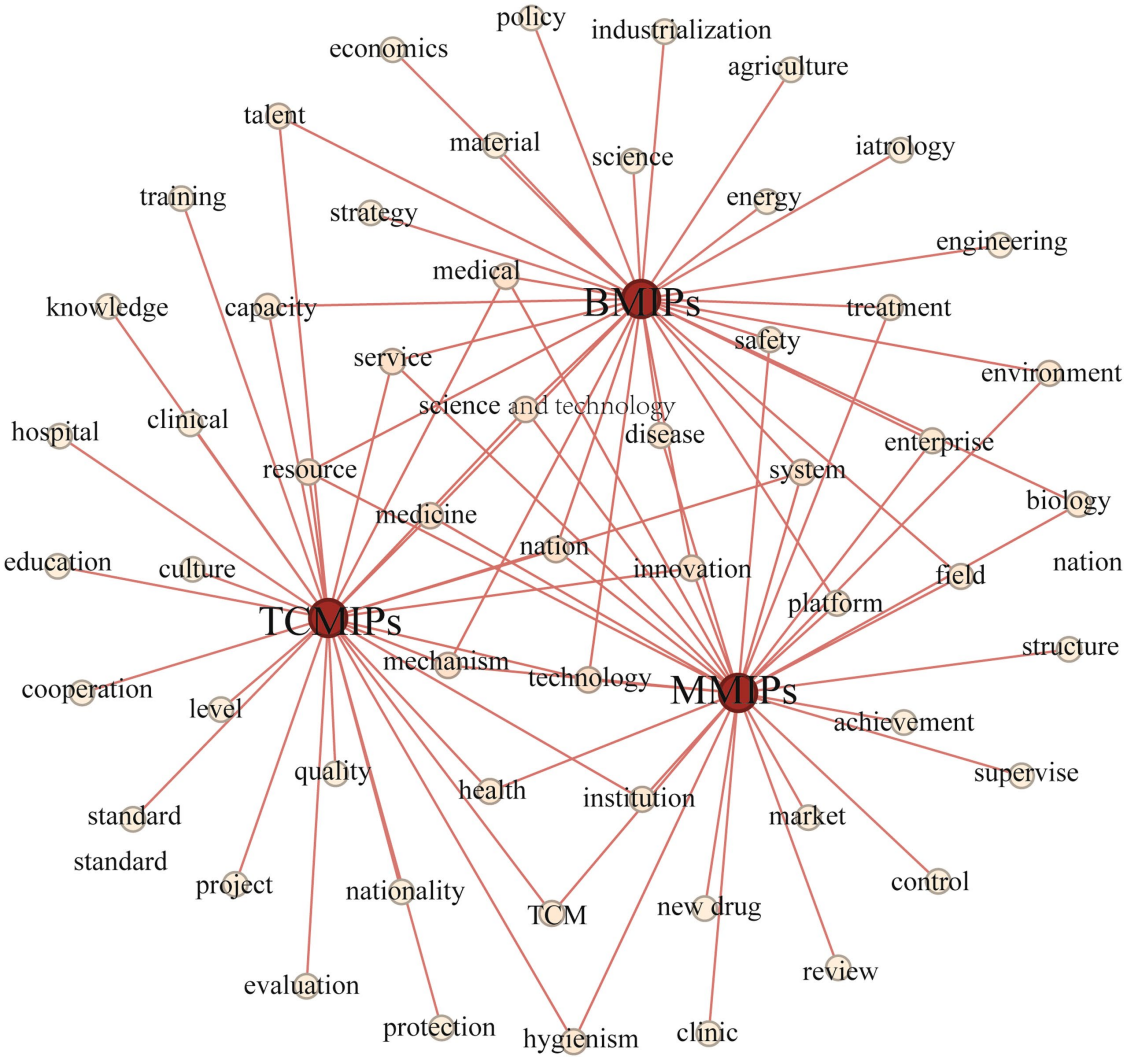
High-frequency words network of TCMIPs, BMIPs and MMIPs.

The central zone surrounded by the high-frequency words reflects the common concern of the TCMIPs, BMIPs, and MMIPs. As shown in Figure 3, it can be seen that “innovation” is located in the center area and has a high frequency, for “innovation” is the core theme of TCMIPs, BMIPs, and MMIPs. Table 5 illustrates that 30% of high-frequency words extracted from the TCMIPs, BMIPs, and MMIPs are the same, with shared highly-frequency words such as “science and technology,” “innovation,” “system,” “mechanism,” and “resource,” suggesting that the common focus of policy is on promoting science and technology development, allocating relevant resources, and improving the medicine innovation system.

**Table 5:** Statistics of the top 30 high frequency words from the TCMIPs, BMIPs and MMIPs.

Serial number	TCMIPs		BMIPs		MMIPs	
	Keywords	Frequency	Keywords	Frequency	Keywords	Frequency
1	TCM	4,742	Biology	1879	Technology	2,670
2	Talent	1,101	Technology	1,156	Medicine	2021
3	Service	1,065	Innovation	378	Innovation	1,106
4	Nation	923	Resource	314	Medical treatment	857
5	Technology	896	Talent	284	Medicine	789
6	Innovation	851	Nation	240	Science and technology	760
7	Health	679	Safety	212	Clinic	709
8	Science and technology	635	Enterprise	204	Hygienism	704
9	Medical treatment	635	Field	181	Enterprise	702
10	Clinical	631	Science and technology	170	Health	565
11	Medicine	600	System	165	Service	544
12	Institution	530	Engineering	149	Biology	499
13	System	528	Capacity	144	TCM	485
14	Project	521	Science	131	Nation	471
15	Hospital	514	Economics	126	System	468
16	Standard	496	Policy	115	Resource	461
17	Nationality	457	Iatrology	115	System	455
18	Knowledge	437	Medical treatment	113	Disease	449
19	Capacity	430	Service	113	Institution	449
20	Mechanism	427	Industrialization	112	Safety	442
21	Resource	422	Energy	103	Review	436
22	Protection	408	Agriculture	103	New drug	360
23	Evaluation	405	Mechanism	102	Platform	344
24	Quality	393	Disease	101	Treatment	321
25	Hygienism	389	Environment	100	Field	317
26	Education	362	Treatment	99	Achievement	313
27	Level	335	Strategy	96	Market	312
28	Culture	324	Platform	95	Mechanism	303
29	Training	318	Medicine	93	Supervise	296
30	Cooperation	312	Materials	93	Environment	295

The TCMIPs and BMIPs all involve high-frequency words such as “talent” and “capability,” indicating that talent cultivation is an important guarantee for the innovation of traditional Chinese medicine and biological medicine. The TCMIPs and MMIPs all involve high-frequency words such as “health,” “clinical,” and “institution,” suggesting that these policies emphasize the clinical efficacy of innovative medicines. The BMIPs and MMIPs all involve high-frequency words such as “enterprise,” “platform,” “treatment,” “disease,” and “safety,” which indicates that pharmaceutical enterprises are encouraged to build innovation platforms to develop efficient and safe innovative medicine. In addition, there is also important information at the edge region in Figure 3, which should not be ignored and reflects the different concerns of TCMIPs, BMIPs, and MMIPs.

The dedicated high-frequency words in TCMIPs involve “nation,” “culture,” “civilization,” “protection,” “criteria,” “evaluation,” and “cooperation,” which indicates that the government emphasizes protection and inheritance for TCMI culture, construction evaluation criteria for TCMI standardized management, and greater international cooperation on TCMI. The dedicated high-frequency words in BMIPs include “economics,” “industrialization,” “strategy,” etc. Due to the small scale of most biopharmaceutical enterprises, they are encouraged to carry out an industrialization strategy to boost BMI development. The dedicated high-frequency words in MMIPs involve “approval,” “regulation,” “achievement,” and “market.” These high-frequency words illustrate that the MMIPs prefer to create a favorable external environment to promote medicine innovation, for example, by speeding up innovative medicine approval, strengthening medicine regulation, encouraging achievement transformation, and cultivating the market environment.

### 4.2 Index analysis and comparison of medicine innovation policy

#### 4.2.1 Index analysis of TCMIPs

Based on the above evaluation system and criteria, we calculate the PMC-Index and determine the level of TCMIPs, as shown in Table 6. The average PMC-Index of 29 TCMIPs is 5.77, which indicates good overall consistency in TCMIPs. Specifically, there are 23 TCMIPs with good consistency and 6 TCMIPs with bad consistency, while there are no excellent and no perfect among the 29 TCMIPs. In addition, these policies are mainly released by the NHC, the NMPA, and the NATCM, suggesting that the Chinese government attaches great importance to TCMI.

**Table 6:** The PMC-Index and level of the medicine innovation policy.

TCMIPs	X_1_	X_2_	X_3_	X_4_	X_5_	X_6_	X_7_	X_8_	X_9_	X_10_	PMC index	Level	Ranking
P1	0.33	0.17	1.00	0.25	0.67	0.33	0.40	1.00	0.75	1.00	5.90	Good	12
P2	0.67	0.83	1.00	0.25	0.67	0.33	0.80	0.60	0.75	1.00	6.90	Good	1
……	……	……	……	……	……	……	……	……	……	……	……	……	……
P28	0.33	0.17	1.00	0.25	0.67	0.67	0.40	1.00	1.00	1.00	6.48	Good	4
P29	0.33	0.17	0.50	0.25	0.33	0.67	0.20	0.40	1.00	1.00	4.85	Bad	27
Average	0.48	0.24	0.88	0.25	0.57	0.38	0.30	0.76	0.91	1.00	5.77	Good	–

BMIPs	X_1_	X_2_	X_3_	X_4_	X_5_	X_6_	X_7_	X_8_	X_9_	X_10_	PMC index	Level	Ranking
Q1	0.83	0.17	1.00	0.25	0.67	0.33	0.20	0.80	0.75	1.00	6.00	Good	8
Q2	0.67	0.17	0.75	0.25	0.67	0.33	0.20	0.60	0.75	1.00	5.38	Good	12
……	……	……	……	……	……	……	……	……	……	……	……	……	……
Q11	0.67	0.17	1.00	0.25	1.00	0.33	0.40	1.00	1.00	1.00	6.82	Good	4
Q12	0.83	0.17	1.00	0.25	0.33	0.33	0.20	0.60	0.75	1.00	5.47	Good	11
Average	0.71	0.18	0.92	0.25	0.72	0.33	0.42	0.85	0.83	1.00	6.21	Good	–

MMIPs	X_1_	X_2_	X_3_	X_4_	X_5_	X_6_	X_7_	X_8_	X_9_	X_10_	PMC index	Level	Ranking
R1	0.67	0.17	1.00	0.25	1.00	0.33	0.60	1.00	1.00	1.00	7.02	Excellent	7
R2	0.50	0.50	1.00	0.25	0.67	0.33	0.20	0.80	0.50	1.00	5.75	Good	22
……	……	……	……	……	……	……	……	……	……	……	……	……	……
R34	0.33	0.17	0.50	0.25	0.33	0.33	0.20	1.00	1.00	1.00	5.12	Good	27
R35	0.67	0.17	1.00	0.25	0.33	0.33	0.40	0.80	0.50	1.00	5.45	Good	24
Average	0.56	0.27	0.93	0.25	0.68	0.37	0.42	0.71	0.87	1.00	6.06	Good	–

With the development of the TCM industry, the focus of TCMIPs has shifted from a general outline (P2, P5) to a specific implementation plan (P20, P21, and P27). For instance, P20 proposes detailed promotion measures for TCM innovation, P21 focuses on medical insurance to support TCM innovation, and P27 emphasizes scientific supervision to stimulate TCM innovation. This reflects the tendency of TCMIP formulations to shift from “rough” to “refined,” which is more conducive to policy implementation (62, 63). It has become one of the strategies emphasized for how to encourage the TCMI in the coming decades.

**Figure 4:**
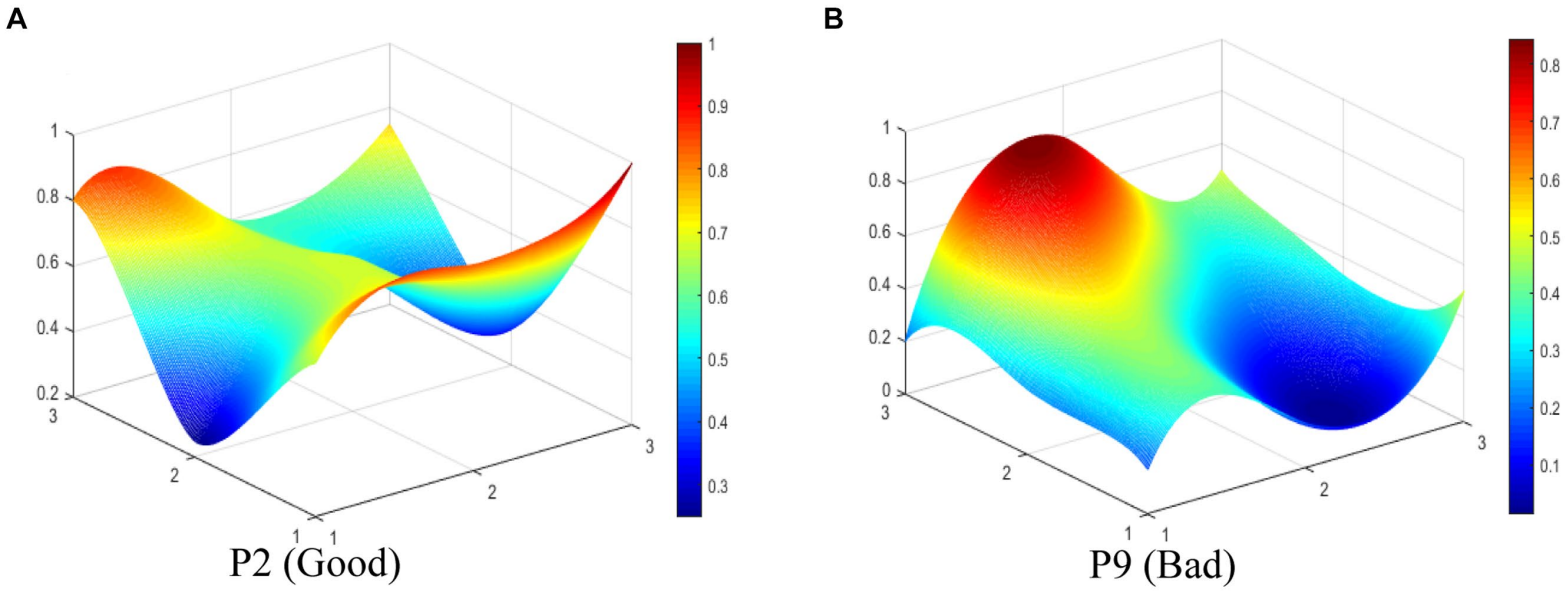
The PMC-Surface of **(A)** P2 and **(B)** P9 of TCMIPs.

In this study, we select P2 (Good level) and P9 (Bad level) to display the differences between TCMIPs visually. The PMC-Surfaces of these selected TCMIPs are drawn according to the PMC matrix (see Figures 4A,B). The convex surface means a higher score on the corresponding primary variable, whereas the concave surface indicates a lower score. The PMC-Index of P2 is 6.90, ranking first among the 29 TCMIPs. P2 is jointly issued by seven government departments, making relatively comprehensive arrangements to promote TCMI. As shown in Figure 4A, the surface shape of P2 is relatively smooth except for the X_6_ (Policy orientation). This is because the policy orientation of P2 only involves encouragement and support, which neglects normative guidance and compulsory requirements. Due to the lower score of P2 on policy orientation, the improvement for P2 should take normative guidance and compulsory requirement on policy orientation into account.

The PMC-Index of P9 is 4.25, ranking 29th among the 29 TCMIPs. As shown in Figure 4B, there is obviously convexity in X8 of P9, while the overall surface shape of P9 is concave compared to P2. This indicates that P9 performs poorly on most of the primary variables. This is due to P9, which measures the registration of scientific and technological achievements in TCM and focuses mainly on the X8 (policy function) but ignores the other primary variables. The improvement path for policy is determined according to the difference between the primary variables and the average (47–49). Thus, the improvement path of P9 is X_9_-X_3_-X_1_-X_5_-X_7_-X_2_-X_6_.

#### 4.2.2 Index analysis of BMIPs

As shown in Table 6, we calculate the PMC-Index and determine the level of BMMIPs based on the above evaluation system and criteria. The average PMC-Index of 12 BMIPs is 6.21, which indicates good overall consistency in BMIPs. Moreover, these policies are mainly released by the SC, the National Development and Reform Commission (NDRC), and the Ministry of Science and Technology (MST), which reflects that the Chinese government attaches much significance to BMI. There are 1 BMIP with excellent consistency and 11 BMIPs with good consistency, while there are no bad or perfect BMIPs among the 12 BMIPs.

Q3 has excellent consistency due to five powerful primary variables: policy object (X_3_), policy instrument (X_5_), incentive method (X_7_), policy function (X_8_), and policy evaluation (X_9_). Q3 is a notice on the biological “11th Five-Year Plan,” which is issued by the SC. This document covers a comprehensive range of policy objects, including government departments, medical institutions, enterprises, and scientific research institutions. Previous studies have divided policy instruments into supply side instruments (SSI), demand side instruments (DSI), and environmental side instruments (ESI) (45, 59). The above three types of policy instruments are used in Q3, including SSI that directly supports BMI development, DSI that directly pulls BMI development, and ESI that creates a favorable external environment for BMI development. Q3 adopts tax benefits, investment subsidies, and intellectual property protection to inspire BMI. The function of Q3 involves five specific aspects of X_8_, which aim to systematically promote innovation in biological medicine. Thus, Q3 is well designed according to five strong primary variables.

**Figure 5:**
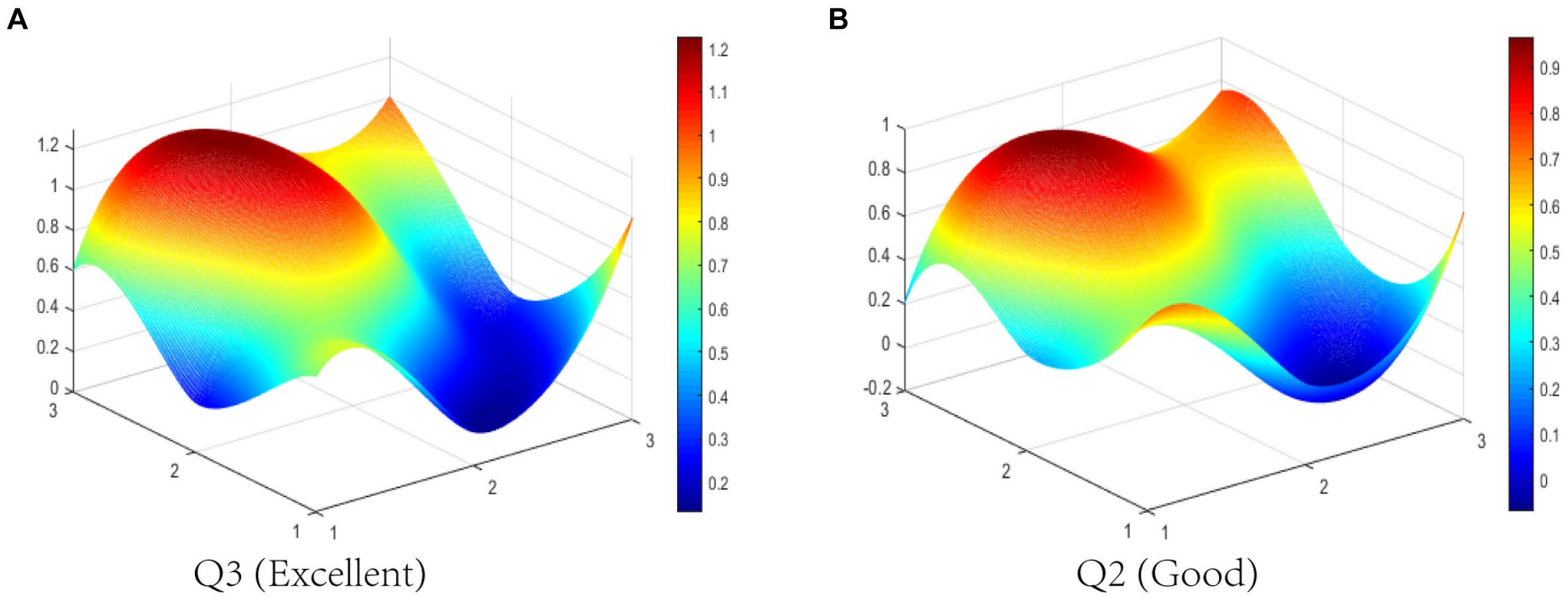
The PMC-Surface of **(A)** Q3 and **(B)** Q2 of BMIPs.

Q3 (Excellent level) and Q2 (Good level) are selected to display the differences between BMIPs visually in this study. The PMC-Index of Q3 is 7.02 ranking first among the 12 BMIPs, while the PMC-Index of Q2 is 5.38 ranking 12th among the 12 BMIPs. The PMC-Surfaces of these selected BMIPs are drawn according to the PMC matrix, as shown in Figures 5A,B. It can be seen that the PMC-Surface of Q3 lies at a higher location than that of Q2, which indicates that Q3 has better consistency. However, Q2 is a special announcement and aims to provide funding for BMI, which is relatively single on the policy scope and has bad consistency. Except for policy timeliness (X4) and policy orientation (X_6_), other primary variables in Q2 are lower than the average in different degrees; thereby, the improvement path of Q2 is X_8_-X_7_-X_3_-X_9_-X_5_-X_1_-X_2_.

#### 4.2.3 Index analysis of MMIPs

We calculate the PMC-Index and determine the level of MMIPs based on the above evaluation system and criteria, as shown in Table 6. The average PMC-Index of 35 MMIPs is 6.06, which indicates good overall consistency in MMIPs. Specifically, there are 9 MMIPs with excellent consistency, 20 MMIPs with good consistency, 6 MMIPs with bad consistency, and no perfect MMIP among the 35 MMIPs. In addition, these policies are mainly released by the SC, the MST, the NHC, and the NMPA, suggesting that there is a higher authority in MMIP policy issuance.

As the medical industry develops, the MMIPs emphasis has shifted from a broad plan (R1, R4) to a detailed incentive scheme (R25, R32, and R34). For instance, R25 focuses on technology transfer to drive medicine innovation; R32 proposes detailed evaluation measures to accelerate innovative medicine to the market; and R34 emphasizes regulatory capacity construction to supervise innovative medicine development. Hence, the current MMIPs focus is to formulate practical and concrete implementation plans to promote medicine innovation.

**Figure 6:**
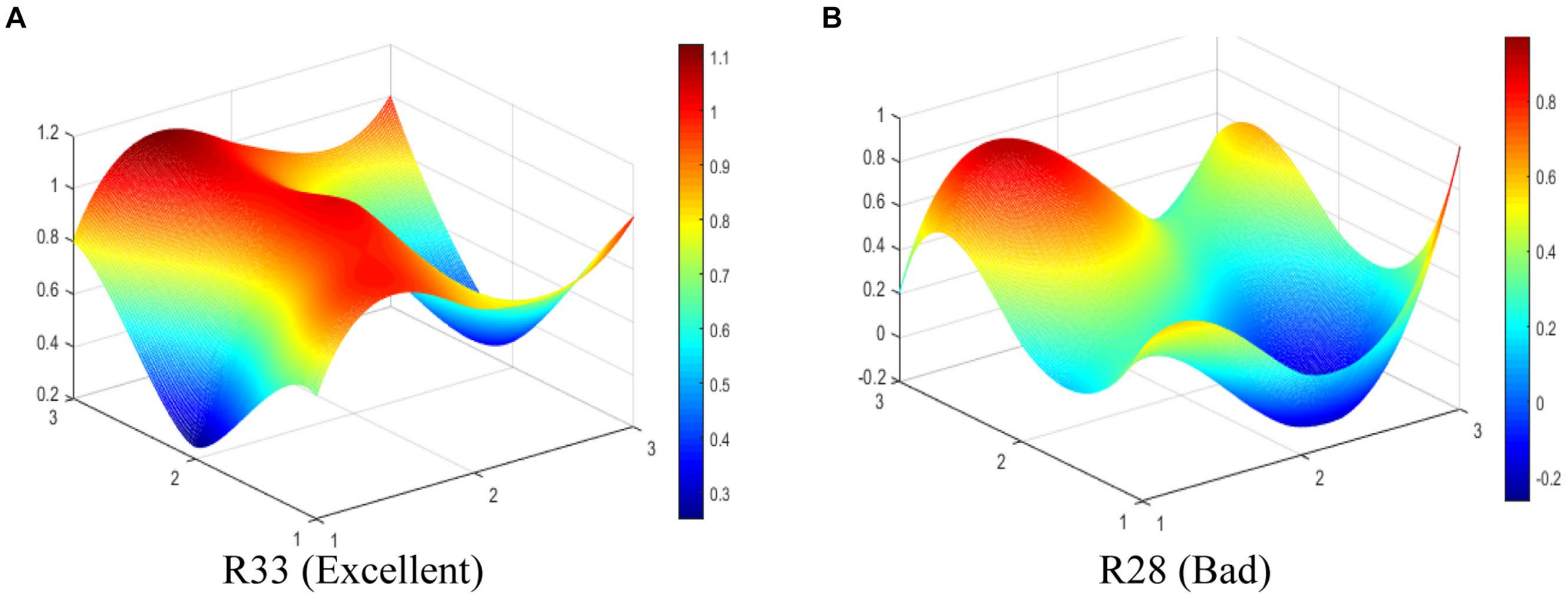
The PMC-Surface of **(A)** R33 and **(B)** R28 of MMIPs.

The R33 (Excellent level) and R28 (Bad level) are selected to display the differences between MMIPs visually in this study. The PMC-Surfaces of these selected MMIPs are drawn according to the PMC matrix (see Figures 6A,B). The PMC-Index of R33 is 7.88, ranking first among the 35 MMIPs. It can be seen that the PMC -Surface of R33 is overall convex, for there are four coordinate points at scale “1” in Figure 6A. This policy is jointly issued by nine government departments, manifesting sufficient coordination and cooperation among departments. R33 aims to clarify the primary goals of medical industry development and accelerate the improvement of the medicine innovation system during the 14th Five-Year Plan period. Overall, R33 has comprehensive content, complete support, and abundant policy instruments, which is a scientifically reasonable policy.

The PMC-Index of R28 is 4.48, ranking 35th among the 35 MMIPs. As shown in Figure 6B, the PMC-Surface of R28 displays fewer convex points, while most surfaces are dented, which suggests that P2 scores lower on various variables. This is because R28 only adopts the means of legal constraints to guide medicine innovation without making specific arrangements in other aspects. The policy issuing agency is single, the policy instrument is deficient, and the policy function is narrow, leading to the dented surfaces of R28. Except for policy object (X_3_) and policy timeliness (X_4_), other primary variables in R28 are lower than the average in different degrees; thereby, the improvement path of R28 is X_8_-X_9_-X_5_-X_7_-X_2_-X_1_-X_6_.

#### 4.2.4 Comparative analysis among TCMIPs, BMIPs, and MMIPs

To clarify the characteristics and differences of various medicine innovation policies in China, we compare the TCMIPs, BMIPs, and MMIPs for further analysis. Upon comparing the average PMC-Index of three types of medicine innovation policies, the order from high to low is BMIPs (6.21) > MMIPs (6.06) > TCMIPs (5.77), which suggests that BMIPs and MMIPs are superior to TCMIPs. In addition, the PMC-Index of TCMIPs range from 4.25 to 6.90, the PMC-Index of BMIPs range from 5.38 to 7.02, and the PMC-Index of MMIPs range from 4.48 to 7.88. In order to clearly display the distribution of the three types of policies across each evaluation level, we create the Table 7. As shown in Table 7, the MMIPs have the highest policy excellence rate (26%), the BMIPs have the highest policy good rate (92%), and the TCMIPs have the highest policy bad rate (21%). Next, we will explore the reasons for the differences through a comparative analysis of primary variables.

**Table 7:** The proportion of TCMIPs, BMIPs, and MMIPs in various levels.

	Perfect	Excellent	Good	Bad
TCMIPs (%)	0	0	79	21
BMIPs (%)	0	8	92	0
MMIPs (%)	0	26	57	17

**Figure 7:**
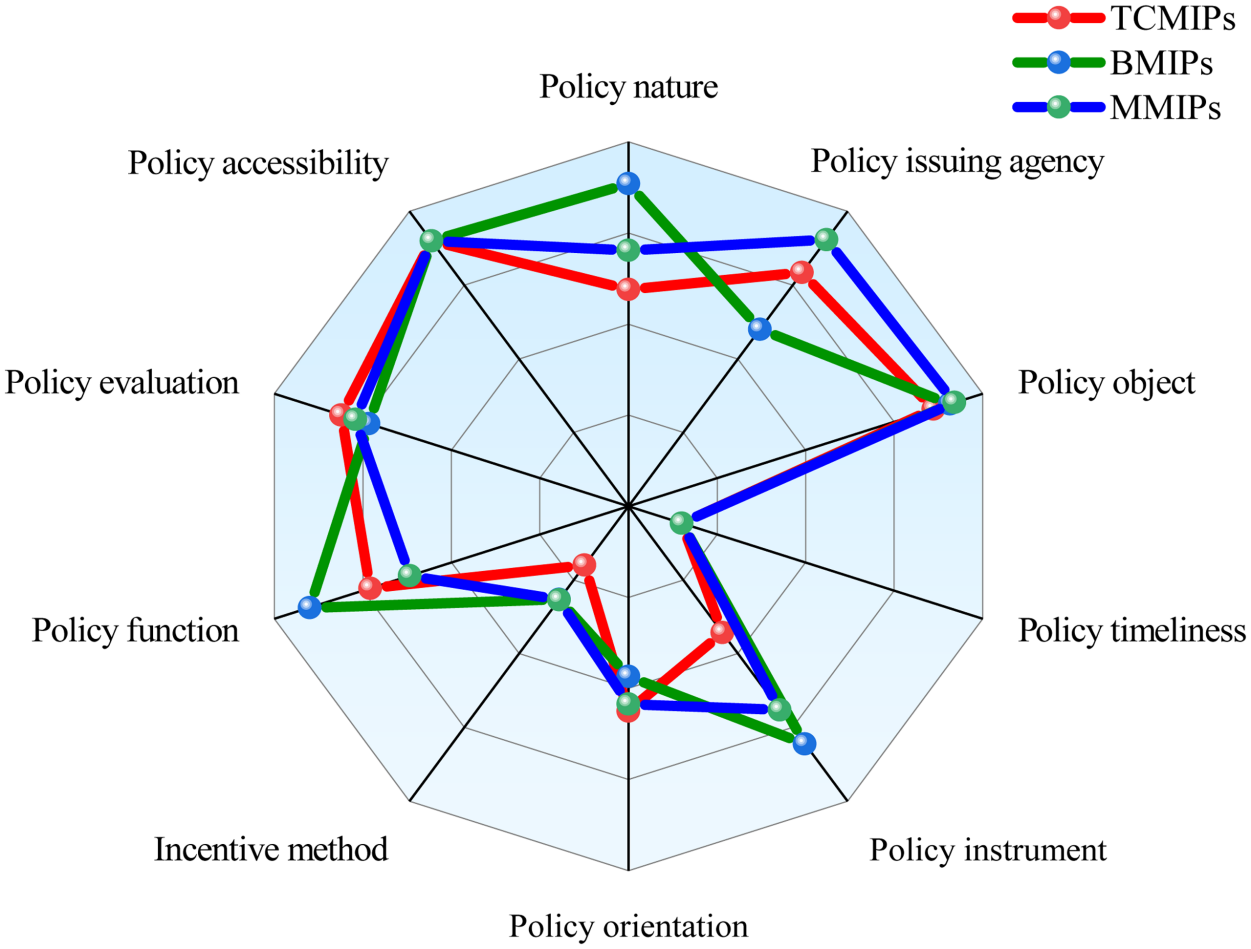
Comparison of primary variables among TCMIPs, BMIPs, and MMIPs.

To show the differences of the various medicine innovation policies more intuitively, we construct a comparative radar chart of the average scores of the primary variables for BMIPs, MMIPs, and TCMIPs. As shown in Figure 7, the BMIPs, MMIPs, and TCMIPs have similar scores on X_3_, X_4_, X_6_, X_9_, and X_10_, while they differ significantly mainly on X_1_, X_2_, X_5_, X_7_, and X_8_. The specific analysis is as follows:

(1) X_1_ (Policy nature). Generally, policy nature is closely related to the purpose of policy formulation; in this paper, policy nature includes prediction, guidance, description, supervision, planning, and support (64, 65). The policy nature of TCMIPs is relatively homogeneous, most only involving guidance and description. Compared with TCMIPs and MMIPs, most BMIPs cover multiple policy natures (i.e., prediction, guidance, description, plan, and support) simultaneously. There are 49% of MMIPs that contain four types of policy natures (i.e., prediction, guidance, description, and plan) at the same time, ranging between TCMIPs and BMIPs. Overall, the three types of medicine innovation policies have less involvement in supervision, which should be incorporated into future policy formulation.
(2) X_2_ (policy issuing agency). The existing studies find that cooperation among policy issuing agencies affects the coordination ability of policy implementation (47, 66, 67). TCMIPs are mainly released independently or jointly by the NHC, the NMPA, and the NATCM, wherein 41% of TCMIPs are released independently by the NATCM and 24% of TCMIPs are released jointly by multiple departments. Moreover, BMIPs are mainly released by the SC, the NDRC, and the MST, whereas only 8% of BMIPs are released jointly by multiple departments. Further, MMIPs are mainly released by the SC, the MST, the NHC, and the NMPA, wherein 54% of MMIPs are released jointly by multiple departments. This suggests that, compared with the BMIPs and the TCMIPs, the MMIPs concentrate more on the coordination and cooperation among departments when formulating policies, which is more conducive to policy implementation.
(3) X_5_ (policy instrument). As mentioned earlier, policy instruments are generally divided into SSI, ESI, and DSI (45, 59). SSI mainly involves the support of financial, infrastructure construction, and technical information. ESI mainly involves regulation, supervision, and public opinion publicity. DSI mainly involves government procurement and service outsourcing. The policy instruments adopted by TCMIPs are SSI and ESI, while BMIPs and MMIPs cover three types of policy instruments. In addition, the usage frequency of DSI in BMIPs is slightly higher than that in MMIPs.
(4) X_7_ (incentive method). Incentive methods refer to measures that promote policy implementation, such as tax benefits, investment subsidies, intellectual property protection, regulatory and evaluation, and administrative approval incentives. From the content of the policy text, some BMIPs and MMIPs adopt multiple incentive methods, while most TCMIPs only involve one incentive method. Thus, in terms of incentive methods, BMIPs and MMIPs are superior to TCMIPs. However, the average score of the incentive methods for BMIPs and MMIPs is 0.42, indicating that they are generally unsatisfactory and that there is much room for improvement.
(5) X_8_ (policy function). The policy function means the social effect that can be achieved after policy implementation (68, 69). In this paper, policy functions mainly include talent cultivation, encouraging innovations, industry-academia-research collaboration, achievement transformation, and international exchange. There are most BMIPs covering the above six functions, followed by TMIPs and MMIPs. It is worth noting that the average policy functions (X_7_) score of MMIPs is 0.71, which indicates that the functions of medicine policies are generally good.

## 5 Discussion

Based on the above analysis results, we will further discuss the following aspects. First, we find that the nature of medicine innovation policy mainly focuses on guidance, description, and plan, which is similar to the findings of existing studies. For example, Zhang et al. (52) researched coal power policy and concluded that these policies are mainly manifested as guidance and description. Yang et al. (13) found that more than 90% of TCM development policies involved guidance and planning. This study, using a larger policy sample, responds to their results. Moreover, we have new findings compared with their research. We find that the nature of supervision is rarely incorporated in medicine innovation policies, and there is a lack of supervision over implementation effectiveness.

Second, there is an overall lack of cooperation among departments in this study from the perspective of the policy issuing agency. This is similar to some previous research findings, which also believe that there is weak collaboration among policy issuing agencies (11, 46, 62). This may be due to differences in specific responsibilities and potential competition among departments, which result in a reluctance to cooperate and coordinate with each other. However, existing research suggests that cross-departmental cooperation is more likely to enhance administrative efficiency (70–72). Moreover, many TCMIPs are independently issued by the NATCM, which is a vice-ministerial department with a relatively low administrative level. These findings are similar to those of Yang et al. (49). In general, the administrative level of the policy issuing agency has a positive impact on the efficiency of policy implementation (66). Furthermore, the two departments that are most relevant to the issuance of medicine innovation policies in China are the NMPA and the NATCM, which are under the State Administration for Market Regulation and the NHC, respectively. Hence, it is difficult to coordinate the management of these two departments and jointly formulate medicine innovation policies in practice.

Third, in terms of policy instruments, BMIPs and MMIPs perform well overall, while TCMIPs need to improve. Specifically, we find that the TCMIPs lack the application of DSI. This paper complements the study of Yang et al. (13), in which policy instruments are not included in the evaluation system. Moreover, Xiong et al. (48) analyzed the application of policy instruments in the fire safety education policy, involving voluntary policy tools, mandatory tools, and mixed policy tools. The classification of policy instrument types differs between Xiong and us, which belong to different schools. Overall, we have further enriched the research on policy instruments.

Finally, the incentive method for the medicine innovation policy is overall insufficient. Specifically, medicine innovation policy in China takes financial subsidies and intellectual property protection as the main incentive methods, while other incentive methods are seldom involved. Similar findings have been reported in previous studies. For example, Yang et al. (49) found that the new energy vehicle policy has inadequate incentives for infrastructure construction. Yang et al. (11) conducted a study on 37 health promotion policies that seldom involve incentives. These findings indicate that insufficient incentives are a common problem with policies. However, a lack of appropriate incentive methods will hinder the rapid growth of the industry.

## 6 Conclusions and implications

### 6.1 Conclusion

This study quantitatively evaluates and analyzes the medicine innovation policies in China since 2000 through mining the text and the PMC-Index model. Specifically, we summarize the respective characteristics of the TCMIPs, BMIPs, and MMIPs and further compare the similarities and differences of the three types of policies from horizontal perspectives. Then, further discussion and analysis are conducted, eliciting suggestions for medicine innovation policy improvement. As far as we know, this study is the first to quantitatively explore the consistency of medicine innovation policies across different types in China and fills the gap in existing literature.

Based on the analysis of the high-frequency words of medicine innovation policy, the results show that the common focus of policy is to promote the development of science and technology, allocate related resources, and improve the pharmaceutical innovation system. In addition, various medicine innovation policies also have their own unique focus. Specifically, the TCMIPs emphasize the inheritance and innovation of TCM; the BMIPs focus on encouraging biopharmaceutical companies to implement industrialization strategies; and the MMIPs tend to create a favorable external environment to promote medicine innovation.

This study utilizes the PMC-Index model to systematically evaluate the TCMIPs, BMIPs, and MMIPs, respectively. The results showed that the average PMC-Index of 29 TCMIPs is 5.77, wherein the policy at the good and bad levels accounts for 79 and 21%, respectively. Compared to BMIPs and MMIPs, the average primary variable values of policy nature (X_1_), policy instrument (X_5_), and incentive method (X_7_) are relatively lower in TCMIPs, so priority could be given to improving TCMIPs in these areas in the future. The average PMC-Index of 12 BMIPs is 6.21, wherein the policy at excellent level accounts for 8% and the policy at good level accounts for 92%, respectively. Moreover, the average PMC-Index of 35 MMIPs is 6.06, wherein the policy at excellent level accounts for 26%, the policy at good level accounts for 57%, and the policy at bad level accounts for 17%, respectively. These results indicate that none of the medicine innovation policies in China reach a perfect consistency level, so there is still room for improvement. In addition, the BMIPs, MMIPs, and TCMIPs have similar scores on X_3_, X_4_, X_6_, X_9_, and X_10_, while they differ significantly mainly on X_1_, X_2_, X_5_, X_7_, and X_8_.

### 6.2 Implications

The theoretical significance of this study is reflected in several aspects. First, this study provides a theoretical reference for the medicine innovation policy formulation in China. In other words, policymakers can design more effective policies by drawing on our study when formulating medicine innovation policies in the future. Second, the PMC-Index model has not previously been used to evaluate medicine innovation policies. Thus, the PMC-Index model is utilized to evaluate the medicine innovation policy in this study, which enriches this stream of literature. Finally, we extend the application context of policy diffusion theory to evaluate medicine innovation policy. Specifically, this paper verifies that the formulation of medicine innovation policy in China conforms to the connotation of policy diffusion theory.

Based on the above analysis, the policy implications proposed in this study are as follows. (1) According to the different policy timeliness, corresponding assessment times should be set when formulating policies, such as long-term monitoring of more than 5 years, medium-term monitoring of 3–5 years, short-term monitoring of 1–3 years, and monitoring within 1 year. Then, establish monitoring standards based on different innovation policy objectives and monitor when the assessment time is reached. Through the establishment of a cross-departmental monitoring information sharing platform, all departments can share their supervision results of medicine innovation policies. Based on the results of supervisory feedback, problems existing in medicine innovation policies should be optimized when formulating policies in the future. (2) The joint superior department of the NMPA and the NATCM can be set up when institutional reform is carried out in the future. This joint superior department plays a connecting role among the SC, other ministerial-level departments, the NMPA, and the State Administration of TCM. The joint superior department can coordinate multiple departments to release medicine innovation policies based on the goals and contents of the policies, improve the administrative level of policy release, and thereby promote medicine innovation. (3) In the formulation of BMIPs and MMIPs, the three policy instruments should be further integrated to bring into play the complementarity among them. Government departments should refer to the application of DSI in BMIPs and MMIPs and incorporate DSI (i.e., government procurement and government purchasing) into the formulation of future TCMIPs, taking into account the characteristics of TCMIPs. DSI can directly stimulate policy receptors and produce significant effects. (4) Incentives should be appropriately enriched and diversified to provide sufficient incentives for medicine innovation in policy formulation. Provide the implementation details of the corresponding incentives, such as the application conditions of the incentive method, to prevent the abuse of the incentives.

### 6.3 Limitations and further research

This study investigates the strengths and weaknesses of medicine innovation policies from the perspective of policy formulation, which provides new insights and a theoretical basis for the evaluation of medicine innovation policies in the future. However, some limitations in our research should be realized. First, there may be some subjectivity in the variable identification. To obtain a more objective and scientific policy evaluation, we can optimize the shortcomings in future research through various methods such as grounded theory, crawler mining, and big data methods. Second, this study mainly selects medicine innovation policies from the national level while not including the local level. In future studies, medicine innovation policies issued by local governments can be included for comparative analysis because they have local characteristics and provide richer references for policy formulation. Finally, this study only evaluates the text of medicine innovation policies in China without analyzing the performance of policy implementation. The difference in difference econometric analysis model can be used to further analyze the implementation effect of medicine innovation policies in the future.

## Data availability statement

The original contributions presented in the study are included in the article/Supplementary materials, further inquiries can be directed to the corresponding author.

## Author contributions

DG: Conceptualization, Data curation, Formal analysis, Investigation, Methodology, Software, Writing – original draft. LQ: Data curation, Formal analysis, Methodology, Supervision, Writing – review & editing. XS: Conceptualization, Data curation, Formal analysis, Funding acquisition, Resources, Supervision, Writing – review & editing.

## Footnotes

^1^ http://www.pkulaw.cn/
